# Maternal androgen excess significantly impairs sexual behavior in male and female mouse offspring: Perspective for a biological origin of sexual dysfunction in PCOS

**DOI:** 10.3389/fendo.2023.1116482

**Published:** 2023-02-15

**Authors:** Nina M. Donaldson, Melanie Prescott, Amy Ruddenklau, Rebecca E. Campbell, Elodie Desroziers

**Affiliations:** Centre for Neuroendocrinology and Department of Physiology, School of Biomedical Sciences, University of Otago, Dunedin, New Zealand

**Keywords:** androgen, PCOS (polycystic ovary syndrome), sexual behavior, DHT (5α-dihydrotestosterone), sexual dysfunction (biological)

## Abstract

**Introduction:**

Polycystic ovary syndrome (PCOS) is the most common infertility disorder worldwide, typically characterised by high circulating androgen levels, oligo- or anovulation, and polycystic ovarian morphology. Sexual dysfunction, including decreased sexual desire and increased sexual dissatisfaction, is also reported by women with PCOS. The origins of these sexual difficulties remain largely unidentified. To investigate potential biological origins of sexual dysfunction in PCOS patients, we asked whether the well-characterized, prenatally androgenized (PNA) mouse model of PCOS exhibits modified sex behaviours and whether central brain circuits associated with female sex behaviour are differentially regulated. As a male equivalent of PCOS is reported in the brothers of women with PCOS, we also investigated the impact of maternal androgen excess on the sex behaviour of male siblings.

**Methods:**

Adult male and female offspring of dams exposed to dihydrotestosterone (PNAM/PNAF) or an oil vehicle (VEH) from gestational days 16 to 18 were tested for a suite of sex-specific behaviours.

**Results:**

PNAM showed a reduction in their mounting capabilities, however, most of PNAM where able to reach ejaculation by the end of the test similar to the VEH control males. In contrast, PNAF exhibited a significant impairment in the female-typical sexual behaviour, lordosis. Interestingly, while neuronal activation was largely similar between PNAF and VEH females, impaired lordosis behaviour in PNAF was unexpectedly associated with decreased neuronal activation in the dorsomedial hypothalamic nucleus (DMH).

**Conclusion:**

Taken together, these data link prenatal androgen exposure that drives a PCOS-like phenotype with altered sexual behaviours in both sexes.

## Introduction

Fertility and sexuality are controlled by the brain and dependent upon complex neuronal circuits that are organized early in life and then activated by sex steroid hormones in adulthood. Prenatal exposure to testosterone, aromatized into oestradiol in the brain, is required for the development of male-typical brain circuitry and behaviors in rodents (1). Over the last 10 years, the dogma that the female brain develops by default in the absence of sex steroids has been challenged with the discovery that peri-pubertal oestradiol and progesterone are also required for feminization of the brain and sexual behaviors (2–5). Noteworthy, the role of androgen signaling through androgen receptor (AR) on the development of the brain and behavior has been recently highlighted in the male through the study of the neural deletion of AR in male mice showing a central role for AR in the development of proper male copulatory behaviors (6, 7). However, the role of AR-mediated signaling remains poorly investigated in the female despite AR being present in the developing female brain and behavior (8). In addition, very little is currently understood about male- and female-typical sexual behaviors that are programmed by *in utero* androgen excess states such as in PCOS (9, 10), congenital adrenal hyperplasia (CAH) (11, 12) and in the case of environmental exposure to androgenic compounds (13–15).

Polycystic ovary syndrome (PCOS) is a common endocrine disorder characterized by androgen excess. PCOS affects roughly 1 in 8 women of reproductive age and is the most common form of anovulatory infertility (16). In addition to hyperandrogenism, PCOS is characterized by menstrual irregularities and polycystic ovarian morphology (16–18) and is associated with a wide range of comorbidities, including obesity, diabetes and cardiovascular disease (16). The hypothalamo-pituitary-gonadal (HPG) axis, that controls fertility and reproductive behavior, is disrupted in many women with PCOS. In particular, luteinizing hormone (LH) pulse frequency, which mirrors gonadotropin-releasing hormone (GnRH) neuron activity and secretion, is significantly elevated. An elevated LH to follicle stimulating hormone (FSH) ratio contributes downstream to polycystic ovarian morphology, elevated androgen production and infertility (19, 20). Steroid hormone feedback to the HPG axis, that would ordinarily slow GnRH/LH secretion is diminished in PCOS patients, suggesting that PCOS originates from a miscommunication between the brain and the ovaries (20–22).

The etiopathogenesis of PCOS is most likely multifactorial with genetic susceptibility and environmental exposure playing predominant roles (9). In line with this, recent studies in men suggest the existence of a male PCOS equivalent in the brothers of women with PCOS (23–26). These men share common endocrine, metabolic and cardiovascular comorbidities with their sisters such as an elevated free androgen index, a low level of FSH leading to an elevated LH/FSH ratio, insulin resistance, type II diabetes and hypertension (23, 25, 26). Among the current hypotheses of PCOS origins, *in utero* androgen excess has been highlighted by human and animal-based study as a substantial contributor (9, 27). Indeed, pregnant women with PCOS show high levels of circulating androgens during gestation, and this is correlated with an increased likelihood of having a daughter diagnosed with PCOS (28). Maternal androgen excess has also been linked to the development of PCOS-like features in a wide range of female mammalian species (27). For example, exposure of female mice to elevated levels of the non-aromatisable androgen dihydrotestosterone (DHT) during late gestation programs the development of hyperandrogenism, irregular oestrous cycles and theca cell hyperplasia (29). These prenatally-androgenized (PNA) female mice exhibit impaired steroid hormone feedback associated with reduced progesterone receptor (PR) expression, elevated LH pulse frequency (29, 30) and elevated GnRH neuronal activity (31), associated with programmed changes in the GnRH neuronal network (29–33). There is some evidence indicating that prenatal androgen excess also alters reproductive function in males. Rams born to mothers exposed to testosterone propionate or dihydrotestosterone exhibit altered testicular function and disrupted neuroendocrine axis function in adulthood (34–38). However, similar disruptions do not appear to be evident in the male siblings of PNA mice modeling PCOS (39).

Epidemiological studies indicate that women with PCOS are more likely to experience sexual dysfunction, including low sex drive and sexual dissatisfaction, that can negatively impact their quality of life (40–48). Interestingly, men diagnosed with early onset androgenetic alopecia (AGA), now considered as a clinical sign of male PCOS equivalent (23, 24, 26), also indicate experiencing sexual dysfunction (49, 50). In women, the cause of PCOS-related sexual dysfunction is frequently attributed to psychological factors, including reduced self-esteem related to hirsutism and/or obesity, a higher prevalence of anxiety, depression and mood disorders and decreased interest in sexual activities due to infertility issues (40–42, 44–46, 48, 51–56). Similarly, in men with AGA, the early onset of baldness is also often discussed in epidemiological studies as a potential factor for sexual dysfunction (49, 50). However, it is not unreasonable to imagine that prenatal androgen exposure that is associated with the programming of PCOS-like reproductive features also might impact the development of male and female sexual behaviors. Obviously, human sexual behavior is incredibly complex and difficult to model, however, the PNA mouse model of PCOS, exposed to the non-aromatisable androgen dihydrotestosterone, provides a powerful reductionist approach to tease apart whether prenatal androgen exposure is associated with changes in sex behavior in male and female mice. To investigate how prenatal androgen excess impacts adult sexual behaviors in female and male mice, we performed a series of sexual behavior tests in PNA male (PNAM) and female (PNAF) mice. Finding a significant impairment in PNAF mice, we then further investigated the potential neural substrates involved in the PNA-related female sexual dysfunction.

## Materials and methods

### Animals

Male and female C57BL/6J mice were generated and housed in the Otago Biomedical Research Facility at the University of Otago until adulthood. Mice were kept under a 12 h light/dark cycle with food and water ad libitum. All mice were kept in same-sex housing from weaning and hence were not exposed to the opposite sex before sexual behavior testing. Adult mice were moved to the Otago Behavioural Phenotyping Unit (BPU) for subsequent behavioral testing. In the BPU, mice were kept under a 12 h reverse light/dark cycle with food and water ad libitum. Sodium lamps permitted observation of the mice during the dark phase. All protocols were approved by the University of Otago Animal Ethics Committee.

### Generation of prenatally-androgenized mice modeling PCOS

Control (VEH) and prenatally-androgenized (PNA) male and female mice were generated using the well-characterized prenatally androgenized (PNA) mouse model protocol (29–33, 57). Adult male and female C57BL/6J mice were paired overnight on the day of proestrus. Gestational day 1 was recorded as the following day after overnight mating and the male was removed from the cage. Females were then monitored for signs of pregnancy such as increased body weight and increased belly circumference. From gestational day 16-19, pregnant dams received a daily subcutaneous (s.c.) injection in the nape of the neck of either 100 µL dihydrotestosterone (DHT, 250 µg/100µL) in sesame oil as the PNA treatment or 100 µL of sesame oil only as the vehicle control. This window of prenatal androgen exposure has been shown to lead to PCOS-like features in mice and largely avoid the critical period for the differentiation of external genitalia (29–33, 57).The male (M) and female (F) offspring of dams injected with DHT (PNAM or PNAF) and vehicle control (VEH) mice were studied from adulthood (postnatal day (PND) 60 onward) in the following experimental protocols. Oestrous cyclicity of VEH and PNA female mice was assessed to establish the expected loss of oestrous cyclicity in PNA mice by collecting daily vaginal smears over a 20-day period (PND 60–80) (**Figure S1**) as previously described (29–31, 33, 58).

### Experiment 1: Phenotyping male and female-typical sexual behaviors in prenatally-androgenized mice

**Figure 1:**
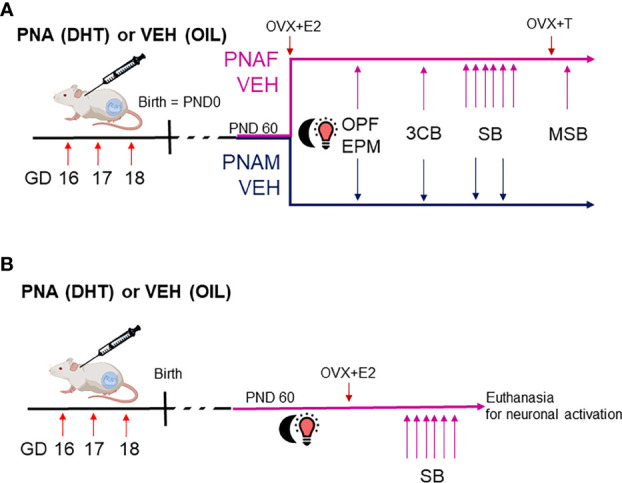
Experimental design to study the effect of prenatal DHT exposure on sexual behaviors in male and female mice. In this study, we used two batches of mice. **(A)** The first batch of mice was used to determine if male and female-typical sexual behaviors could be affected by prenatal androgen exposure (experiment 1 and 2). Dams were treated with either dihydrotestosterone (DHT) or oil vehicle (VEH) at gestational day (GD) 16, 17 and 18. After birth, the male and female offspring entered the experimental protocol at adulthood around postnatal day 60 (PND60). PNAF and VEH females (magenta line) were ovariectomized and implanted with a silastic capsule containing estradiol in order to normalize hormonal status and artificially induced receptivity (OVX+E2). PNAM and VEH male mice (blue line) were left intact. Then, the animals entered a series of behavioral procedures to test their anxiety-like behavior and locomotion within an open-field (OPF) and an elevated-plus maze (EPM) tests, their abilities to discriminate a sexual partner within a three-compartments box partner preference test (3CB) and their typical sexual behavior (SB, i.e. lordosis behavior for female over 6 consecutive weeks and male copulatory behavior within 2 consecutive weeks). Finally, the PNAF and VEH female mice had their estradiol implant replaced by an implant containing testosterone (OVX+T) in order to activate and therefore test male-like sexual behavior (MSB) and verify if prenatal DHT exposure could have led to masculinization of the brain. **(B)** The second batch of mice was used to identify the neural target of prenatal androgen in female mice associated with impaired lordosis behavior. Only the female offsprings were used in this experiment 3. The PNAF and VEH female mice underwent ovariectomies and implantation of a silastic capsule of estradiol followed by 6 consecutive weeks of lordosis behavior testing. At the last test, PNAF and VEH female mice were euthanized 90 min after the beginning of the test in order to study neural activation induced by lordosis behavior. VEH, control male; PNAF, prenatally androgenized female; PNAM, prenatally androgenized male; PND, postnatal day; OPF, open-field test; EPM, elevated plus-maze; 3CB, three-chamber test; SB, sexual behavior test; MSB, male-like sexual behavior.

A cohort of C57Bl6 male (n=9-12/group) and female (n=6-11/group) control and prenatally-androgenized (PNA) mice underwent the following behavioral tests as previously described (3–5) (**Figure 1**).

### Anxiety and locomotion tests

Male and female-typical sexual behavior in mice are highly impacted by anxiety and dependant upon locomotor activity. Since previous studies in the PNA mouse model highlighted anxiety-like behaviors during the diurnal/inactive phase (59–61), we decided to test the basal level of anxiety and locomotion in PNA mice under the same conditions used for testing sexual behavior: during the nocturnal/active phase and under a sodium light imperceptible by mice. Mice were tested in Open-field tests and Elevated-plus maze tests to determine basal locomotor activity and anxiety as detailed below.

*Open-field test:* Mice were placed in the center of a plexiglass aquarium (40 x 40 x 30 cm) under sodium lamps and their movements were recorded for 10 min. Between each animal, the aquarium was cleaned using 10% ethanol. Video recordings were analyzed using TopScan^®^ software. Two virtual zones of the aquarium floor were demarcated: the center zone (60% of the total floor area) and the periphery zone (surrounding area). TopScan^®^ recorded the distance travelled by the mice and the amount of time mice spent in each zone over 10 min (600 s). Locomotion was measured as the distance travelled (mm). Anxiety behavior was determined by comparing the time (s) spent in the center versus peripheral zone, with less time in the center reflecting heightened anxiety.

*Elevated-plus maze:* The maze apparatus was comprised of four arms (30 cm long, 5 cm wide) elevated 40 cm above the ground by metal legs and arranged in a plus shape. Two arms were open without walls and two arms were enclosed by high walls (15 cm). Mice were placed at the junction of the four arms (center) and their movements were recorded for 10 min. Between each animal, the maze was cleaned using 10% ethanol. Video recordings were analyzed using TopScan^®^. Each of the four arms and the center zone were virtually demarcated. The distance travelled by the mice and the amount of time spent in each arm and center over the 10 min (600 s) period was traced and recorded. Locomotion was measured as the distance travelled (mm) and anxiety behavior was measured by the time (s) spent in the center, the open arms and the closed arms, with less time in the open arms reflecting heightened anxiety.

### Ovariectomies and hormones replacement for induction of female receptivity

All female mice in this study were ovariectomized and implanted with a silastic capsule of oestradiol as previously described (3–5). This ensures uniform hormone concentrations across females and mimics an oestrus hormonal level of estradiol in order to artificially trigger receptivity (3–5). Briefly, all females were bilaterally ovariectomized under general anaesthesia with isofluorane. At the same time, a 5-mm-long Silastic capsule (inner diameter: 1.57 mm; outer diameter: 2.41 mm) containing crystalline 17β-oestradiol (E2758, Sigma-Aldrich, USA) (diluted 1:1 with cholesterol (C8667, Sigma-Aldrich, USA)) was inserted under the skin at the nape of the neck to induce oestrous levels of oestradiol. Mice received Carprofen (5 mg/kg) and were allowed to recover for two weeks before the onset of behavioral tests. On the day of testing, the females (either tests or stimuli) were administered progesterone (P) (P0130, Sigma-Aldrich, USA) (500 µg/mL, s.c.) 3 hours prior to test commencement.

### Male and female partner discrimination test

*Three-Chambers Box Partner preference:* A partner preference test was carried out in virgin animals prior to sexual behavior experiments. Partner preference testing was conducted in a plexiglass box divided into three compartments (60 x 13 x 30 cm) by opaque partitions with fenestrations that allow for odours to diffuse throughout the arena. The day before testing, test females were allowed to habituate in the central compartment for 10 min. On the day of testing, females were administered P (500 µg/mL s.c.) 3 h prior to test commencement. For the test, two other mice, an intact sexually experienced stimulus male and an oestrous stimulus female (OVX + E2 female injected with P 3 h prior to the experiment, OVX+E+P) were placed in the two lateral compartments with their own bedding in order to make their respective compartments as odorous as possible. The test animal (PNAM/PNAF or VEH counterpart), was then placed in the middle compartment containing no bedding and observed for 10 min (the experimenter was blinded to group at the time of experiment). The time (s) that the test female spent actively sniffing each partition was recorded with a stopwatch. Between each test, the middle compartment was cleaned using 10% ethanol to eliminate the previous test subject’s odours. A preference score was calculated by dividing the time spent investigating the male compartment minus the time spent investigating the female compartment by the total time spent investigating both compartments. A positive value of the preference score indicates a partner preference toward the stimulus male, whereas a negative value of the preference score indicates a partner preference toward the stimulus female.

### Male sexual behaviors

All male mice were gonadally intact and sexual behavior tests were conducted in a transparent plexiglass aquarium (35 x 25 x 19 cm) filled with a layer of fresh sawdust as previously described (3–5). At the beginning of each test, the male was placed alone in the cage and allowed to adapt for 15 min. A receptive female (OVX+E+P) was then introduced into the cage and the latencies to the first mount and intromission, the latency to ejaculate, as well as the number of mounts, intromissions, and pelvic thrusts, were recorded. The test lasted until ejaculation occurred or 30 min if no ejaculation was achieved. If a male never displayed a certain behavior within the 30 min test, the latency was scored as 1800 s.

### Female sexual behaviors

*Female typical lordosis behavior:* The lordosis behavior test was carried out over six consecutive weeks allowing seven days of rest between each test. Lordosis behavior testing was conducted in the same Plexiglas aquarium as described above. For the test, a sexually experienced male was placed in the aquarium and allowed to habituate for 15 min. Subsequently, a test female was introduced to the aquarium and the pair was observed. The number of mounts exhibited by the male and the number of lordosis behavior displays exhibited by the female was recorded for 15 min. The lordosis quotient corresponds to the number of lordosis postures recorded following a trial mount by the male, divided by the number of mounts attempted by the male throughout the duration of the test, and multiplied by 100 (i.e. (number of lordosis/numbers of male mount trials) * 100).

*Anogenital sniffing/aggressive behaviors:* During all lordosis behavior tests, bouts of anogenital investigation and aggressive behavior toward the male were counted. Bouts of anogenital sniffing was recorded every time the female nose was in contact with the male genitalia. Bouts of aggressive behaviors were recorded every time the female attempted to kick or to bite the male.

### Experiment 2: Determining if prenatal androgen excess drives masculinized sex behavior

Following the female typical sexual behavior tests, a random subset of VEH female (n = 6) and PNAF (n = 5) mice were anesthetized again with isofluorane in order to remove the oestradiol implant, and replaced it with a testosterone implant as previously described (4). The testosterone implant was made of a 5-mm-long Silastic capsule (inner diameter: 1.57 mm; outer diameter: 2.41 mm) filled with crystalline testosterone (T1875, Sigma-Aldrich, USA) diluted 1:1 with cholesterol (C8667, Sigma-Aldrich, USA). This procedure mimics typical testosterone levels of adult male mice by 2 weeks and can induce male-like sexual behavior in female mouse (4). Mice received Carprofen (5mg/kg) and were allowed to recover for 10 days before additional behavior testing. Following recovery, male-like sexual behavior (i.e. mounting, intromission and pelvic thrust) was tested in a plexiglass aquarium (35 x 25 x 19 cm) filled with a layer of fresh sawdust as previously described (62). Briefly, a test female (PNAF or VEH) was placed alone in the aquarium to habituate for 15 min. Subsequently, an oestrous stimulus female (OVX + E + P) was introduced to the aquarium and the pair was observed. The initial latency to mount, number of mounts and number of pelvic thrusting movements shown by the test female (PNAF or VEH) were scored over 30 min.

### Experiment 3: Mapping sex behavior-related neuronal activation in prenatally-androgenized mice

A second cohort of VEH female (n=5) and PNAF (n=5) mice were tested for lordosis behavior as described above over 6 consecutive weeks. After the last lordosis test, i.e. week 6, female mice were euthanized 90 minutes after the beginning of the lordosis test in order to study neuronal activation following lordosis behavior. In addition, we also euthanized a group of adult females C57Bl6 mice (Basal n=3-4) who went through the same procedure as the VEH and PNAF mice except that no male stimulus was introduced into the aquarium for the last lordosis behavior test. This basal group allowed us to determine the basal neural activation without lordosis behavior.

### Tissue processing for immunostaining

Upon completion of all behavioral tests, female mice were anesthetized with a lethal i.p. injection of pentobarbital (150 mg/kg/mice) and perfused transcardially with 4% cold paraformaldehyde. Brains were removed and post-fixed in 4% paraformaldehyde overnight. Brains were then cryoprotected in 30% sucrose/tris-buffered saline (TBS) solution over 72 h. Free-floating brain sections were cut at 30µm-thickness on a freezing microtome and collected in cryoprotectant. Forebrains were cut coronally from the rostral telencephalon to the posterior hypothalamus. Sections were collected in four different series and stored at -20°C until immunostaining.

### Immunohistochemistry procedures

All the following immunohistochemistry procedures were performed on brain sections from the second cohort of female VEH (n=5) and PNAF (n=5) mice after lordosis behavioral testing. One set of sections (i.e every fourth section) was rinsed for 10 minutes in TBS six times to remove cryoprotectant. Sections were then treated with 3%H_2_O_2_ and 40% methanol in TBS for 10 minutes to quench endogenous peroxidases, and then washed a further three times in TBS. Sections were incubated in blocking solution with 2% normal donkey/goat serum and 1% bovine serum albumin in TBST (TBS + 0.3% Triton-X) for 1 hour. Sections were then incubated with primary antibodies against Kisspeptin, Progesterone Receptor and cFOS (**Table 1**) for 96 hours at 4 degrees. Then, the sections were washed again three times 10 minutes before incubation with IgG biotinylated secondary antibodies (donkey anti-sheep IgG biotinylated/goat anti-rabbit IgG biotinylated) diluted in blocking solution for 90 minutes at room temperature. Sections were then washed before being incubated with the avidin-biotin complex diluted in TBST (1/200, ABC, Vector Laboratory, Burlingham, CA). To finish, after rinsing, sections were reacted with 3,3’diaminobenzidine tetrahydrochloride in TBS with nickel ammonium sulfate and glucose oxidase. After a last wash, the sections were mounted onto gelatin-coated slides, dried for 48h, dehydrated in ethanol followed by xylene and then coverslipped with DPX.

**Table 1:** Primary antibodies used.

Proteins of interest	Primary antibodies	Dilutions
Kisspeptin	#Kp052, INRAe	1:10000
Progesterone	#63605, Abcam	1:800
cFOS	#sc-52, Santa Cruz	1:1000

### Image analysis

Photomicrographs of cFOS and PR immunohistochemistry in different brain regions were captured using a 20x objective on a bright field microscope (Olympus BX51). The representative images of each brain region analyzed were identified using the mouse brain atlas from Paxinos and Franklin, 3rd Edition. An experimenter blinded to treatment counted the number of cFOS- and PR-immunoreactive nuclei using Image J software®.

For the kisspeptin immunohistochemistry, photomicrographs of the anteroventral periventricular nucleus (AVPV) were captured using a Nikon Eclipse TiE2 inverted microscope at x20 magnification. Z-stacks of 1um focal thickness were captured across three representative sections of the AVPV. The images were then analyzed by a blinded experimenter using the NIS-element software (RRID : SCR_014329). The experimenter counted the number of kisspeptin immunoreactive cells on three representative sections of the AVPV.

### Statistical analysis

Statistical analysis was performed with PRISM^®^ software 9.0 (Graph Pad Prism, RRID:scr_002798). Normal distribution and homogeneity of variance were determined using a Shapiro-Wilk test and Fisher’s test, respectively. The percentage of animals performing either male sexual behaviors or female typical lordosis behavior were compared by a Fisher exact tests. All data are represented as the mean +/- SEM. Analysis of lordosis, investigative and aggressive behaviors was performed by a 2-way ANOVA mixed models for repeated measures. All other data were analyzed by an unpaired Students t-tests when the normal distribution and variance homogeneity parameters were met. Otherwise, a Mann-Whitney test was used. A p-value < 0.05 was considered statistically significant and a p-value <0.07 was considered a tendency.

## Results

### Experiment 1: Male and female-typical sexual behaviors are impaired in prenatally-androgenized mice

#### Male sexual behavior is slightly altered by prenatal androgen exposure

**Figure 2:**
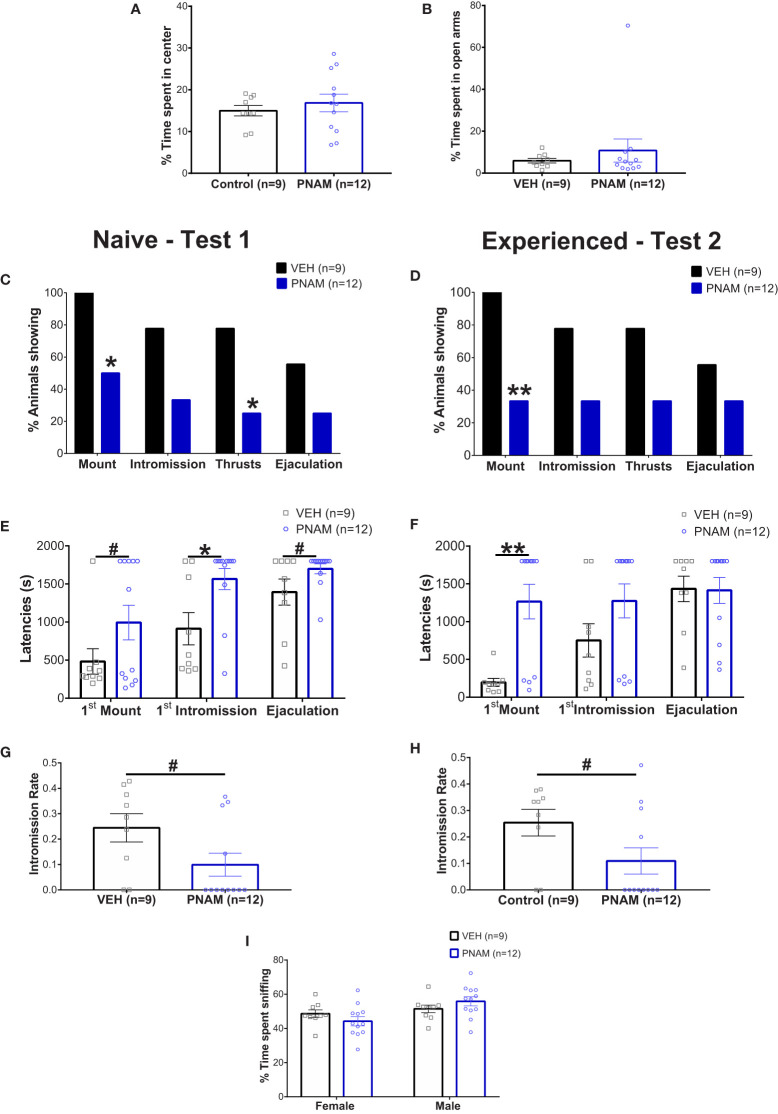
Prenatal DHT exposure modifies male-typical sexual behavior. **(A, B)** Histogram representing the percentage of time spent in the center zone of the Open-field test **(A)** and the percentage of time spent in the open arms of the Elevated-plus maze **(B)** for the VEH male (black bar) and PNAM (dark blue bar). **(C, D)** Histograms representing the percentage of animals performing each component of male copulatory behavior (mount, intromission, pelvic thrusts and ejaculation) while naive **(C)** (i.e. during the first test) or experienced **(D)** (i.e. during the second test) for the VEH male (black bars) and PNAM (dark blue bars). **(E, F)** Histograms representing the latencies to first mount, first intromission and ejaculation while naive **(E)** (i.e. during the first test) or experienced **(F)** (i.e. during the second test) for the VEH male (black bars) and PNAM (dark blue bars). **(G, H)** Histograms representing the intromission rate, corresponding to the number of mounts with intromission divided by the total number of mount trial, while naive **(C)** (i.e. during the first test) or experienced **(D)** (i.e. during the second test) for the VEH male (black bar) and PNAM (dark blue bar). **(I)** Histograms representing the percentage of time spent sniffing either the male of the female compartment in the three-compartment box partner preference test for the VEH male (black bars) and PNAM (dark blue bars). **p<0.01, *p<0.05, ^#^p<0.07. VEH, control male; PNAM, prenatally androgenized male, s, seconds. Mean +/- SEM.

Locomotion was not affected by prenatal androgen excess in male, indicated by both the open-field test (10155 +/- 652.8 mm for VEH and 9318 +/- 554.4 mm for PNAM; t=0.98, df=19, p=0.34) and the elevated-plus maze test (6663 +/- 367.9 mm for VEH and 7004+/-279.6 for PNAM; U=52, p=0.92). Basal anxiety was also not affected by prenatal DHT exposure, as indicated by the percentage of time spend in the center of the open-field (**Figure 2A**; t=0.697, df=19, p=0.49) and in the open-arm of the elevated plus maze (**Figure 2B**; U=52, p=0.92).

Two sexual behavior tests were performed because male sexual behavior is subject to learning with an increase in sexual behavior efficiency after a first experience (63, 64). In the first test, 55% of VEH males exhibited complete sexual behavior with mounting, intromission, pelvic thrust and ejaculation (**Figure 2C**). In contrast, only 25% of PNAM exhibited complete sexual behavior (**Figure 2C**). Significantly fewer PNAM performed mounting (p=0.02) or thrusts (p=0.03) compared to VEH males (**Figure 2C**), while the percentage of animals performing intromission (p=0.08) and ejaculating (p=0.20) by the end of the first test was not statistically different (**Figure 2C**). After sexual experience, in test 2, 55% of VEH males and 33% of PNAM exhibited complete sexual behavior (**Figure 2D**). While the percentage of PNAM performing mounting behavior remained significantly reduced compared to VEH males (p=0.0046, **Figure 2D**), the percentage of animals exhibiting intromission (p=0.08), thrusts (p=0.08) and ejaculation (p=0.39) were not different between PNAM and VEH during the second test.

To further investigate the effect of prenatal DHT exposure on male sexual behavior, we analyzed different components of male typical sexual behavior such as the latencies to perform (**Figures 2E, F**) and numbers (**Table 2**) of each behavior were also measured. In the first test, we observed that the latency to first intromission increased significantly for the PNAM compare to the VEH (**Figure 2E**; t=2.68, df=19, p=0.03). In addition, we observed a tendency to an increase latency to mount (t= 1.704, df=19, p=0.07) and to ejaculate (t=1.841, df=19, p=0.07) for the PNAM compared to their VEH counterparts (**Figure 2E**). These increased latencies are associated with significantly fewer mounts with intromission (U=27, p=0.0467) and fewer pelvic thrusts (U=25, p=0.0251) in PNAM compared to VEH males (**Table 2**). In addition, the intromission rate of the PNAM tended toward being decreased compare with VEH males (**Figure 2G**; U=28.5, p=0.057). During the second test, only the latency to first mount was significantly increased for the PNAM compared to the VEH males (**Figure 2F**; t=3.972, df=19, p=0.001). PNAM and VEH had the same latencies to first intromission (t=1.621, df=19, p=0.13) and ejaculation (t=0.08, df=19, p=0.65) during the second test. Interestingly, PNAM displayed significantly fewer mounts (U=13, p=0.0018), mounts with intromission (U=23, p=0.0185) and pelvic thrusts (U= 24, p=0.0233) compared to the VEH males (**Table 2**) which was also associated with a trend toward a lower intromission rate (**Figure 2H**; U=28, P=0.052) during the second test.

**Table 2:** Prenatal DHT exposure affect the number of different parameters of male copulatroy behvaiour in the adult male mice. PNAM, prenatally-androgenized male. Mean +/- SEM.

*Number of*	Naive Male (Test 1)		Experienced Male (Test 2)	
	*Control (n=9)*	*PNAM (n=12)*	*Control (n=9)*	*PNAM (n=12)*
Mounts	13.22 +/- 3.227	8.417 +/- 3.607	22.67 +/- 5.649	3.667 +/- 1.982**
Mounts with Intromission	6.889 +/- 2.664	2.417 +/- 1.598*	10.67 +/- 3.078	2.25 +/- 1.415*
Pelvic Thrusts	153.2 +/- 55.46	58.42 +/- 36.14*	211 +/- 65.71	54.58 +/- 26.08*

**p-<0.01, *p-<0.05.

#### Male partner discrimination is not affected by prenatal androgen exposure

Male sexual behavior is dependent upon the odor recognition of a partner. Therefore, the ability of male mice to recognize a female was assessed using the three compartments box partner preference tests. PNAM and VEH male mice spent a similar percentage of time sniffing the male (p=0.4117) and the female (p=0.4116) compartments (**Figure 2I**). Noteworthy, the total time spent sniffing both compartments was also not different between PNAM and VEH male mice (413 +/- 10.45 s for VEH and 389.25+/- 10.91 s for PNAM; t=1,529, df=19, p=0.22).

#### Female sexual behavior is significantly impaired by prenatal androgen exposure

**Figure 3:**
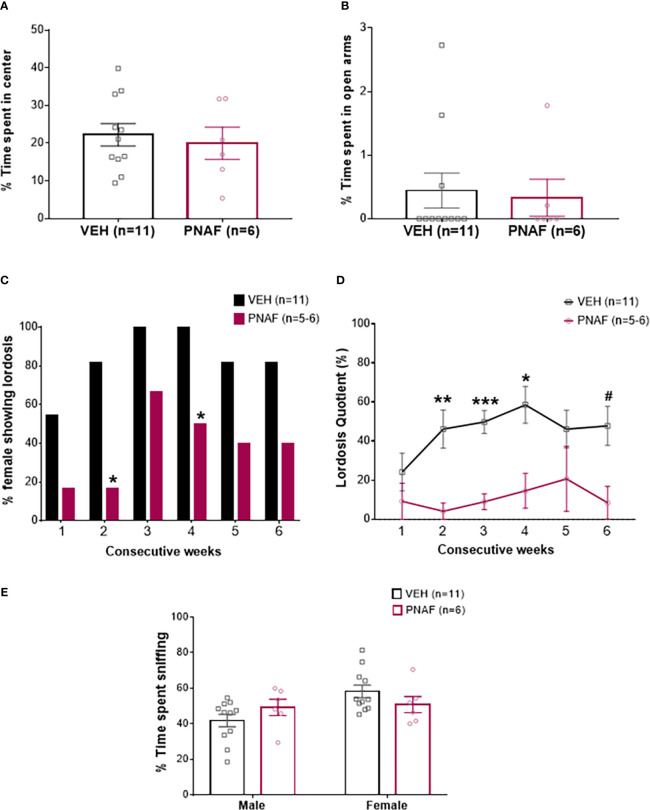
Prenatal DHT exposure significantly impairs female-typical sexual behavior in the prenatally-androgenized female mouse modeling PCOS. **(A, B)** Histograms representing the percentage of time spent in the center zone of the Open-field test **(A)** and the percentage of time spent in the open arm of the Elevated-plus maze **(B)** for the VEH female (black bars) and PNAF (magenta bars). **(C)** Histograms representing the percentage of animals showing lordosis behavior for the VEH female (black bars) and PNAF (magenta bars) over 6 consecutive weeks of testing. **(D)** Graphical curve representing the percentage of lordosis quotient, i.e. the number of lordosis behavior divided by the number of male mount trial, over the 6 consecutive weeks of testing for the VEH female (black line) and PNAF (magenta line). **(E)** Histograms representing the percentage of time spent sniffing either the male or the female compartment in the three-compartment box partner preference test for the VEH female (black bars) and PNAF (magenta bars). ^#^p<0.06, *p<0.05, **p<0.01, ***p<0.001. VEH, control female; PNAF, prenatally androgenized female. Mean +/- SEM.

Before to test for the female typical sexual behavior, lordosis, we needed to verify that prenatal androgen exposure was not altering locomotion and anxiety in the PNAF compare to control female mice (VEH). Surprisingly, we observed a slight increase of the distance travelled by PNAF compare to VEH female mice in the open-field test (5336 +/- 438.7 mm for VEH and 6923 +/- 944.7 mm for PNAF; U=13, p=0.048) while no effect of prenatal DHT exposure on locomotion was observed in the elevated-plus maze test (2199 +/- 151.8 mm for VEH and 2436 +/- 330.4 for PNAF; t=0.75, df=15, p=0.46). Despite this discrepancy in the locomotion, basal anxiety, represented by the percentage of time spend in the center of the open-field (**Figure 3A**; t=0.44, df=15, p=0.67) and in the open-arm of the elevated plus maze (**Figure 3B**; U=32, p=0.96), was not affected by prenatal androgen exposure.

The female typical sexual behavior, lordosis, is a learned process. Therefore, we tested lordosis behavior over 6 consecutive weeks. As expected, during the first three weeks, the percentage of VEH female mice exhibiting lordosis behavior increased to reach 100% and then remained over 80% until the last week of testing. In contrast, the percentage of PNAF mice showing lordosis behavior remained lower throughout the 6 consecutive weeks of testing compared to VEH females: 16.67% at week 1 and 2, 66.67% at week 3, 50% at week 4 and only 40% at week 5 and 6. The percentage of PNAF displaying lordosis behavior was significantly reduced compare to VEH females at week 2 (p=0.03) and 4 (p=0.03) (**Figure 3C**). These results were associated with a significant reduction in the lordosis quotient in PNAF mice compared to VEH females (**Figure 3D**). Statistical analysis with repeated measures mixed model ANOVA revealed a significant effect of the prenatal androgen treatment (F (1,15) = 25.60, p=0.0001). Following *post hoc* comparisons, the lordosis quotient was significantly reduced in PNAF mice compared to VEH females in week 2 (*p=0.0091*, t=3.988, df=13.14), week 3 (*p=0.002*, t=5.731, df=14.99) and week 4 *(p=0.0271*, t=3.383, df=13.75). For the last test in week 6, the *post-hoc* comparison detected a trend toward a decreased lordosis quotient in PNAF compared to VEH females (*p=0.0583*, t=3.016, df=12.95).

#### Investigative and rejection behaviors during lordosis

Sexual motivation was assessed by quantifying the number of times the female sniffed the anogenital region of the male: anogenital sniffing bouts (**Table 3**). Prenatal androgen treatment had no significant effect on the number of anogenital sniffing bouts (F(1,15) = 0.01529, *p=0.9032*).

**Table 3:** Prenatal DHT exposure does not affect investigative and aggressive behaviors in the adult female mice. Mean +/- SEM. W, week; PNAF, prenatally-androgenized female. Mean +/- SEM.

	Anogenital Sniffing bouts		Aggressive behaviors bouts	
	Control (n=11)	PNAF (n=5/6)	Control (n=11)	PNAF (n=5/6)
**W1**	0.30 +/- 0.20	1.17 +/- 0.65	3.0 +/- 0.75	5.17 +/- 1.51
**W2**	1.45 +/- 0.62	1.50 +/- 0.56	3.45 +/- 1.36	4.0 +/- 1.81
**W3**	1.91 +/-0.72	1.50 +/- 0.50	0.91 +/- 4.17	4.17 +/- 1.80
**W4**	0.73 +/- 0.36	1.00 +/- 0.63	2.36 +/- 0.89	1.67 +/- 0.84
**W5**	1.18 +/- 0.40	1.00 +/- 0.55	1.09 +/- 0.74	3.40 +/- 1.40
**W6**	2.55 +/- 0.79	2.20 +/- 0.86	2.64 +/- 0.86	7.20 +/- 2.69

Rejection behaviors toward the male were assessed by the number of times the female rejected male approaches by kicking or biting (**Table 3**). Prenatal androgen treatment was found to have no significant effect on the number of aggressive behavior bouts (F (1,15) = 3.296, *p=0.0895*).

#### Female partner discrimination is not affected by prenatal androgen exposure

The female-typical sexual behavior, lordosis behavior is also dependent upon the odor recognition of a male partner. Therefore, the ability of mice to recognize the male was assessed using the three compartments box partner preference tests. PNAF and VEH female mice spent a similar percentage of time sniffing the male (p=0.39) and the female (p=0.39) compartments (**Figure 3E**). The total time spent sniffing both compartments was not different between PNA and VEH female mice (332.81 +/- 18.16 s for VEH and 328.60 +/- 29.09 s for PNA; U=18, p=0.30).

### Experiment 2: Prenatal androgen exposure with DHT, a non-aromatisable androgen, does not masculinize sexual behavior in female mice

**Figure 4:**
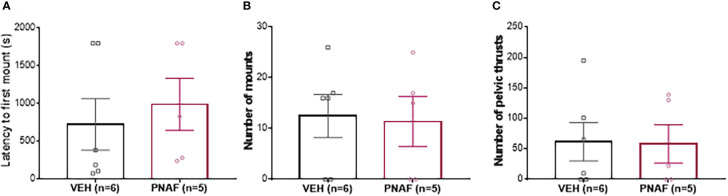
Prenatal DHT exposure does not masculinize behavior in the prenatally-androgenized female mouse modeling PCOS. **(A)** Histogram representing the latency to first mount for VEH female (black bar) and PNAF (magenta bar). **(B)** Histograms representing the number of mounts for VEH female (black bar) and PNAF (magenta bar). **(C)** Histograms representing the number of pelvic thrusts for VEH female (black bar) and PNAF (magenta bar). VEH, control female; PNAF, prenatally androgenized female. Mean +/- SEM.

To determine whether prenatally androgenized females exhibited masculinized sexual behaviors, adult PNAF and VEH female mice were subjected to elevated testosterone which is triggers male-like sexual behaviors in presence of a receptive stimulus female (**Figure 4**). The latency to first mount was not different between PNAF (991.4 +/- 346 s) and VEH (726.0 +/-342.4 s) female mice (U=10, *p=0.3853*) (**Figure 4A**). The number of mounts performed by the female over an oestrous stimulus female was also not statistically different between PNAF (11.40 +/- 4.95 mounts) and VEH (12.50 +/- 4.24 mounts) female mice (t=0.1699, df=9, *p=0.8688*) (**Figure 4B**). Finally, the number of pelvic thrust-like movements performed by the female during mount of the stimulus female was also not different between PNA (58.20 +/- 31.44 pelvic thrusts) and VEH (61.83 +/- 31.42 pelvic thrusts) female mice (t=0.810, df=9, *p=0.9372*) (**Figure 4C**).

### Experiment 3: Identifying female sexual behavior-related neural changes in prenatally-androgenized mice

#### AVPV Kisspeptin neuron population size is not affected by PNA exposure

AVPV Kisspeptin neurons have been demonstrated to play a major role in the expression of lordosis behavior (65). In addition, AVPV kisspeptin neurons are sexually dimorphic (66) and sensitive to prenatal testosterone rise (67). Here, we found that the number of kisspeptin immunoreactive cells per section of the AVPV was not different between PNA (7.27 +/- 1.35 kisspeptin positive cells) and VEH (8.74 +/- 1.28 kisspeptin positive cells) female mice.

#### Progesterone receptor immunoreactivity is not different between VEH and PNA female mice after priming with ovarian hormones (OVX+E+P)

**Figure 5:**
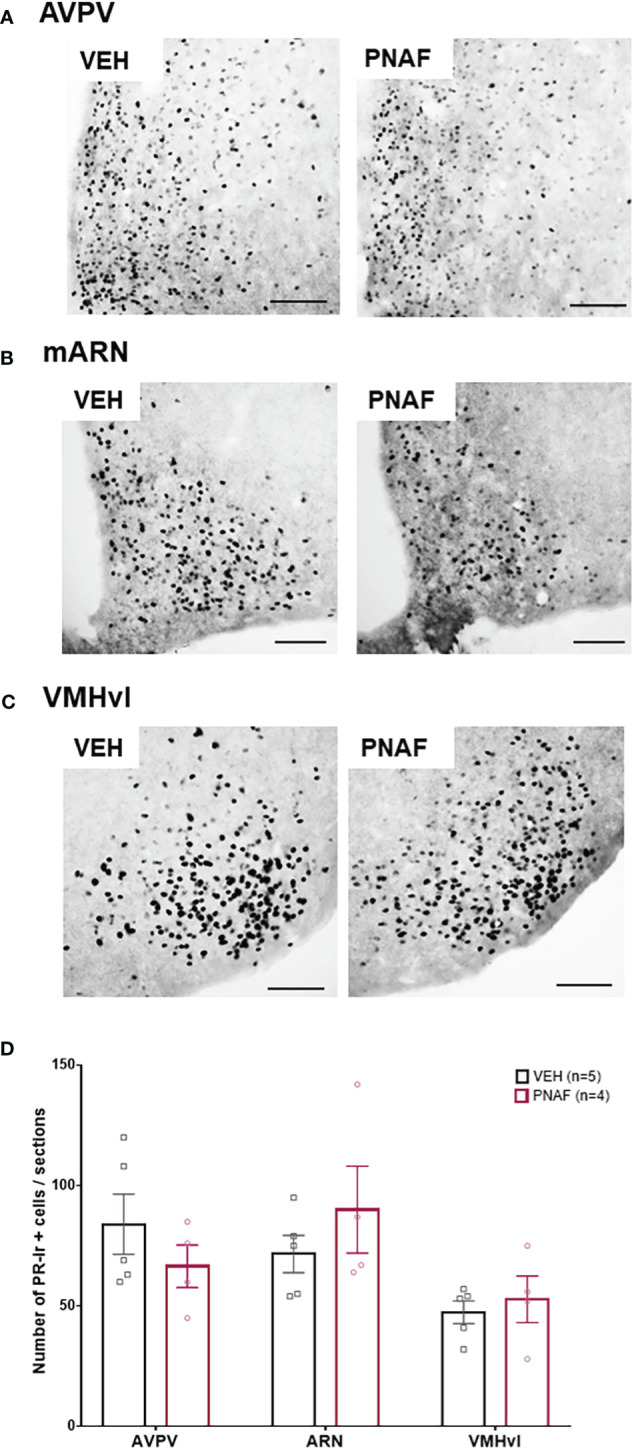
Prenatal DHT exposure does not alter adult progesterone receptor expression in after hormonal normalization. **(A–C)** Representative photomicrographs of PR immunoreactivity in the anteroventral part of the periventricular nucleus of the third ventricle **(A)**, the medial arcuate nucleus **(B)** and the ventrolateral part of the ventromedial hypothalamus **(C)** in VEH and PNA mice. **(D)** Histograms representing the number of Progesterone receptor (PR) immunoreactive cells per sections analyzed between ovariectomized and hormone replaced PNAF mice (magenta bars) and VEH female controls (black bars) within the three brain regions analyzed. AVPV, anteroventral part of the periventricular nucleus of the third ventricle; ARN, arcuate nucleus; VMHvl, ventrolateral part of the ventromedial hypothalamus. VEH, control female; PNAF, prenatally androgenized female modeling PCOS. Mean +/- SEM. Scale bar: 100μm.

In adulthood, lordosis behavior is dependent upon oestradiol and progesterone signaling in the brain (68–70). As reduced PR immunostaining has been reported in intact PNA mice (30), we aimed to determine if reduced lordosis behavior observed in PNA females might correspond with reduced PR expression in brain regions regulating female sexual behavior. PR-positive cells were counted in the anteroventral periventricular nucleus (AVPV), the median arcuate nucleus (mARN) and the ventrolateral part of the vendromedial hypothalamus (VMHvl) (**Figures 5A–D**). Robust PR-immunoreactivity was observed in both groups throughout the regions analyzed and no differences were found in the number of PR-positive cells in any of the regions analyzed between PNA and VEH mice that had previously been OVX and steroid primed for sexual behavior analysis (**Figures 5A–D**).

#### Neural activation is reduced only in the dorsomedial hypothalamus after lordosis behavior in prenatally-androgenized female mice

**Figure 6:**
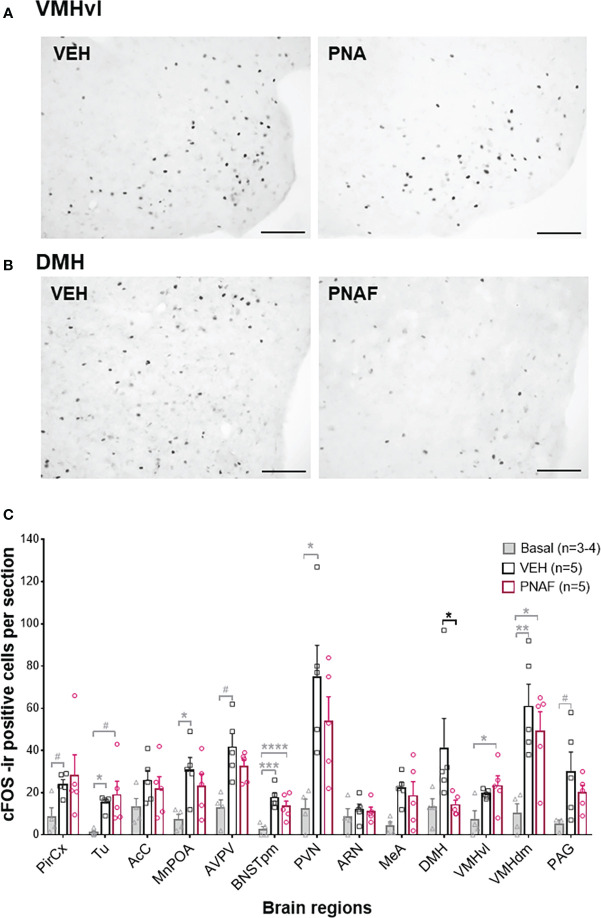
Prenatal DHT exposure decreases neural activation only in the dorsomedial hypothalamus of the PNAF modeling PCOS **(A, B)** Representative photomicrographs of cFOS immunoreactivity in the ventrolateral part of the ventromedial hypothalamus (VMHvl) **(A)** and the dorsomedial hypothalamus (DMH) in VEH and PNA mice. **(C)** Histograms representing the number of cFOS immunoreactive cells per section analyzed for each brain regions studied for Basal female who did not undergo lordosis behavior (grey bars), VEH control females (black bars) and PNAF (magenta bars) who were tested for lordosis behavior during the 6^th^ test. Mean +/- SEM. *p<0.05, Mann-Whitney test. VEH, controls; PNA, prenatally androgenized; Pir Cx, piriform cortex nuclei; Tu, olfactory tuberal nucleus; AcC, Accumbens nucleus core; MnPOA, median preoptic area; AVPV, anteroventral part of the periventricular nucleus of the third ventricle; BNSTpm, posterior median part of the bed nucleus of the stria terminalis; PVN, parventricular nucleus of the hypothalamus; ARN, arcuate nucleus; DMH, dorsomedial hypothalamus; VMHvl, ventrolateral part of the ventromedial hypothalamus; VMHdm, dorsomedian part of the ventromedial hypothalamus, MeA, medial amygdala; VTA, ventral tegmental area; PAG, periaqueductal nucleus. # p<0.07, * p<0.05, **p<0.01, ***p<0.001, ****p<0.0001. The grey bars and grey statistical signs represent the differences between the Basal and VEH groups while the black bars and black statistical signs represent the differences between the PNAF and VEH groups. VEH = control female; PNAF= prenatally androgenized female modeling PCOS. Mean +/- SEM. Scale bar: 100μm.

In a second cohort of PNA and VEH female mice, that were tested for lordosis behavior again during 6 consecutive weeks, we detected again a significant impairment of lordosis behavior in PNA female mice over the 6 consecutive tests (F (1,8) =13, *p=0.0069*). Ninety minutes following the last lordosis behavior test, animals were euthanized and neural activation was assessed by cFOS immunostaining in several brain regions known to be implicated in sexual behaviors and/or fertility regulation in female mice (**Figures 6A–C**). As expected, the number of cFOS-positive cells increased between control females that did not participate in lordosis behavior (Basal, n=3-4) and control females who underwent lordosis behavior tests (VEH, n=5) (**Figure 6A**). Indeed, a significant increase in the number of cFOS-ir positive cells was observed between the Basal and VEH female mice in the majority of the brain regions analyzed and known to be implicated in the regulation of lordosis behavior: the olfactory tuberal nucleus (TU; p=0.03), the median preoptic area (MnPOA; p=0.03), the posterior median part of the bed nucleus of the stria terminalis (BNSTpm; p=0.0009), the paraventricular nucleus of the hypothalamus (PVN; p=0.01) and the dorsomedian part of the ventromedial hypothalamus (VMHdm; p=0.007) (**Figure 6A**). In addition, we found a trend toward an increase in cFOS-ir positive cells between Basal and VEH female mice in three other regions: the Piriform Cortex (PirCx; p=0.06), the anteroventral part of the periventricular nucleus of the third ventricle (AVPV; p=0.06) and the periaqueductal gray nucleus (PAG; p=0.067) (**Figure 6A**). In contrast, the numbers of cFOS-ir positive cells were not different between PNAF and VEH mice in the majority of brain regions analyzed except for the dorsomedial hypothalamus where we counted significantly lower cFOS-ir positive cells in PNAF mice (14.2 +/- 2.25) compared to VEH female mice (41 +/- 14.15) (U=1, *p=0.0159*) (**Figures 6A, C**).

## Discussion

This study aimed to investigate how maternal androgen excess impacts adult sexual behavior in male and female offspring. We demonstrate here, that prenatally androgenized female mice that model several PCOS features (29–33, 57–61) and their male siblings exhibit altered adult sexual behaviors. We found that PNAM displayed reduced performance in some components of sexual behavior, mostly the mounting and intromission behavior parameters. However, PNAM were able to perform complete sexual behavior similarly to control males independently of their sexual experience. In contrast, PNAF exhibited impaired lordosis behavior throughout the 6 weeks of testing. In addition, female PNA mice displayed similar male-like sexual behaviors to VEH female mice after hormonal replacement with testosterone to induce male-like sexual behavior. This result demonstrates that the observed sexual dysfunction in PNAF is not likely due to masculinization of the brain. This finding is supported by the AVPV kisspeptin population size remaining unaffected in PNAF mice. These results suggest, instead, a specific impairment in the feminization of the brain in the PNA mouse model of PCOS. Noteworthy, in our study condition with PNA and VEH female mice being ovariectomized and supplemented with artificial high level of oestradiol, the PR expression is similar between the PNAF and VEH in three brain regions known to regulate lordosis behavior. This result suggests that the sexual dysfunction of the PNA female mice could have an organizational origin. Lordosis behavior-induced cFOS expression was largely unchanged, but apparently reduced activation of the DMH suggests potential avenues for future investigation. Interestingly, the sexual dysfunction experienced by women with PCOS and men with AGA has long been dismissed as an unfortunate symptom of impaired body image and self-esteem (42). Our findings suggest an alternative theory regarding the origins of PCOS-related sexual dysfunction. These data in an animal model indicate programming effects of prenatal androgen excess on the regulation of adult sexual behavior. The effect of maternal androgen excess appears to vary from a pernicious effect for the male offspring to a detrimental effect for the female offspring, leading to an inability to copulate with a male. These findings may be applicable beyond PCOS, to other diseases where androgen signaling is enhanced from early life such as in congenital adrenal hyperplasia (CAH) (11, 12). Moreover, exposure to endocrine disrupting chemicals may lead to increased androgen production or androgen receptor activity at early stages of life (13–15).

### Prenatal androgen exposure in male mice altered male copulatory behavior but does not alter their fertilization capabilities.

To date, most of the studies on the role of androgens on male copulatory behavior have focused on the role of testosterone, aromatized into estradiol and therefore acting on estradiol receptors for the development but also the activation of male sexual behavior (1). Studies on the role of androgens acting through the androgen receptor remains sparse (6). A set of recent studies using androgen receptor and estradiol receptors deletion within the central nervous system in mice highlighted that neural androgen receptor signaling throughout life is required for the full expression of male copulatory behavior (7, 71, 72). Studies in sheep injected with either testosterone propionate or DHT during gestation indicate that maternal androgen excess compromises the reproductive function of ram offspring (34–38, 73) without altering sexual behavior (38). Here, we investigated for the first time, the effect of prenatal exposure to the non-aromatisable androgen, DHT. We observed an alteration only in certain sexual behaviors parameters. Indeed, naïve and experienced PNAM needed more time to perform their first attempt to mount and intromit the stimulus female. They also performed fewer mount with and without intromission as well as fewer pelvic thrusts. These results could suggest a lack of motivation for socio-sexual interaction which remains to be determined. Interestingly, at the end of both tests, a similar number of PNAM and control males were able to reach ejaculation, the endpoint of male copulatory behavior. In addition, the PNAM were more similar to VEH males after experience with only the mounting behavior remaining significantly reduced during the second test. These findings are aligned with previous work demonstrating no significant impairments in the neuroendocrine hypothalamo-pituitary-gonadal axis in PNAM mice (39). Altogether, these findings suggest that while prenatal DHT exposure disrupts some parameters of adult male copulatory behavior, mostly motivational components, the endpoint capability of the PNAM to fertilize a female mouse is unaffected. Similarly, sexual dysfunction in men with androgenic alopecia, a clinical sign of PCOS equivalent in men, has been also associated with motivation such as decreased desire and decreased sexual arousal without affecting erectile function (50).

### Prenatal androgen exposure in mice specifically impairs lordosis behavior

In females, PNA resulted in a sustained impairment in lordosis behavior. In an attempt to decipher the cause of this sexual dysfunction, several behaviors related to sexual behavior that might explain the observed reduction of sexual receptivity in the PNA mouse were also examined. Anxiety and the physical ability to perform sexual behaviors are considered to be potential confounding factors that might influence an animal’s ability to perform normal sexual behaviors. PCOS patients are more likely to develop anxiety and depression which have been shown to negatively impact their quality of life (41, 45, 51–54). It was therefore pertinent to determine changes in anxiety and locomotion evident in the PNAF mouse model. Previous studies have reported anxiety-like behaviors in the PNA mouse model of PCOS (59–61). However, here, no significant differences were detected in anxiety-like behaviors in PNAF mice. This discrepancy is likely to be experimental as the previous studies were performed during the light phase of the light-dark cycle i.e. the inactive phase of the animals. In the present experiment, tests were performed under a sodium lamp, which cannot be seen by mice, during the dark phase of the light-dark cycle i.e. the active phase of the animals. Those conditions are the same conditions that were used to test lordosis behavior, partner preference and male-like sexual behavior. Therefore, the decrease in lordosis behavior observed in the PNAF mice is unlikely to be due to basal anxiety or deficits in locomotion during their naturally active phase. However, we cannot rule out that the introduction of the male stimulus could have triggered an anxiety-like response similar to the one observed when the animals where tested during their inactive phase in the previous studies (59–61).

Like lordosis, male-oriented sexual partner preference is a female-typical. Behavioral tests found no overt difference in the partner preference of the PNAF mice compared to VEH females, suggesting that prenatal androgen does not impact preference for male or female scent. Ano-genital sniffing and defensive behaviors were also unchanged in PNAF, suggesting that reduced sexual motivation and/or an increase in aggression toward the male are unlikely to explain the PNA related sexual dysfunction.

In agreement with other studies, repeated testing of lordosis yielded a steady increase in the lordosis quotient over time in VEH females (3–5). PNAF mice, however, did not exhibit this same increase in lordosis quotient over time. Further investigation is needed to determine if prenatal androgen exposure modifies the neuroplasticity occurring in the brain to learn lordosis behaviors (74, 75) or other cognitive behavioral outcomes such as learning, memory, or social interactions.

Impaired sexual behaviors have also been reported in the female offspring of dams exposed to anti-mullerian hormone (prenatal AMH or PAMH mice) (76), a paradigm that also models PCOS features (77). In addition to impaired lordosis, PAMH females also demonstrated altered partner preference and increased rejection/aggression behaviors (76). Differences in partner preference and aggression behaviors between the two PCOS-like models may reflect the nature of the androgen exposure. PAMH likely drives elevated maternal testosterone, which when aromatized in the fetal brain will result in masculinization (1).

### Prenatal DHT does not masculinize the female brain and behavior

How excess non-aromatisable androgens like DHT impact the development of the female brain and behavior remains poorly understood (78) despite androgen receptor being present in the female brain (6). Here, we determined that prenatal androgenization with DHT that models PCOS does not masculinize the female brain or sex behaviors. The levels of three domains of male-like sexual behaviors (latency to mount, mounting behavior and pelvic thrusting) were indistinguishable between PNAF mice and control females. These behavioral findings were supported by anatomical data demonstrating a feminized AVPV kisspeptin population as already described (79). AVPV kisspeptin neurons are a clearly sexually differentiated population in the brain, with the number of neurons decreased by the male-typical prenatal testosterone rise (67).

### Organizational versus activational effects of sex steroid hormones?

Oestradiol and progesterone are crucial for the expression of female-typical rodent sexual behavior (69). In PCOS patients, progesterone and estradiol levels can be abnormal in association with impaired folliculogenesis and ovulation. To overcome differences in circulating oestradiol and progesterone levels between PNA and VEH, animals were ovariectomized and the hormones required for lordosis behavior were replaced. This also effectively removed the adult hyperandrogenism observed in this model (29), and allowed us to investigate the potential organizational effects of prenatal exposure to DHT on sexual behaviors. Impaired lordosis in PNA mice following a normalization of circulating steroid hormones, therefore, suggests an earlier, organizational impact of sex steroids or other downstream signals on the neural substrates controlling lordosis behavior. The organizational effect of oestradiol and progesterone for feminization of the brain has been demonstrated to occur during the peripubertal period (3–5). As hormone replacement in the present study occurred in adults, after the expected rise in endogenous testosterone levels in the PNA model (at 40-50 days of age) (33), we cannot rule out an impact of this pubertal rise in androgens on the circuits mediating female-typical sexual behavior.

PCOS is associated with impaired estradiol and progesterone feedback to the reproductive axis to slow GnRH/LH secretion (21, 80, 81), suggesting a central insensitivity to steroid hormone signaling. Knowing that PNA mice also model this impaired steroid hormone feedback to the reproductive axis by exhibiting mainly a reduced number of PR expressing cells in several hypothalamic nuclei (29, 30), we investigated whether impaired lordosis may be the result of an impaired ability to respond to the artificially delivered hormones (implant of estradiol and injection of progesterone). PR was robustly expressed and not different between VEH and PNAF mice in any of the areas investigated, suggesting that exogenous hormone treatment together with the absence of hyperandrogenism can overcome the impaired progesterone feedback observed in the intact PNAF mice (29), and that this is not a likely explanation for impaired lordosis behavior.

### Which neural target could explain the lordosis behavior impairment?

In an effort to identify the neural target of prenatal DHT exposure correlated with lordosis behavior impairment, cFos immunoreactivity, a proxy for neural activation, was measured after lordosis behavior in a wide range of brain regions known to be implicated in the neuronal circuitry controlling female sexual behaviors (68). Although cFos expression was significantly elevated in all of the expected regions in mice experiencing lordosis compared to a basal control group, cFos expression patterns were largely unaffected by PNA. In view of the recent evidences highlighting the role of RP3V Kisspeptin and VMHvl nNOS neurons in lordosis behavior in healthy female mice (65) as well as in the PAMH mouse model of PCOS (76), we would have expected some changes in cFOS in the RP3V and VMHvl region. As noted earlier RP3V Kisspeptin neurons remain unchanged in the PNA mice (data showed here and recently published (79). Similarly, a previous study from our group showed no changes in arcuate nucleus nNOS neurons in the PNA mice contrary to the PAMH mouse model (76). Finally, our data showed an absence of cFOS and PR changes in the VMHvl in the PNA mouse model compare to control therefore it is unlikely that VMHvl nNOS neurons could be involved in the sexual dysfunction observed in the PNA mouse model of PCOS. The changes in Kisspeptin and nNOS observed in the PAMH likely results from a masculinization of the brain circuit controlling sexual behavior since prenatal AMH treatment leads to increase testosterone level (77). Noteworthy, our cFOS data highlighted a change in the DMH, with PNAF showing reduced cFOS immunoreactivity compare to controls suggestive of reduced activation in this area. The role of the DMH in female sexual behaviors remains unclear, however, RF-amide related-peptide 3 (RFRP-3) neurons in the DMH have recently been identified as potential novel factors of female sexual motivation and behaviors in addition to their known roles in energy balance and GnRH neuron function (82). Interestingly, recent studies also showed that injection of RPRP-3 leads to suppression of either sexual motivation or receptivity in female hamsters, rats and eusocial mammals (83–85). Thus, further investigation on RFRP-3 in the PNAF mice would be of interest to decipher the potential role of RFRP-3 in the suppression of lordosis behavior.

## Conclusion

These new data provide a significant step forward in our knowledge of how prenatal androgenization can influence adult sexual behaviors in male and female mice. These findings are aligned with the “Developmental Origins of Health and Diseases” (DOHaD) hypothesis in which early-life environment can increase sensitivity or risk toward developing adverse outcomes later in life. Combined with evidence that prenatal androgen exposure leads to reproductive disorders in both sexes, these findings suggest a critical sensitive period of development where both the neuroendocrine regulation of reproductive function and behavior are sensitive to androgen specifically through the androgen receptor. The underlying central mechanisms underpinning impaired sexual behavior in PCOS-like female mice and their male siblings remains to be elucidated.

## Data availability statement

The raw data supporting the conclusions of this article will be made available by the authors, without undue reservation.

## Ethics statement

The animal study was reviewed and approved by University of Otago Ethical committee.

## Author contributions

ND, as the first author, was a honours student under the supervision of ED (co-last author). She performed most of the experiments under ED supervision. MP is an ARF within the Campell Laboratory. She provided her technical assistance throughout the study as well as provided feedbacks on the manuscript. AR is a PhD student working on progesterone receptor changes in PCOS under supervision of ED and RC has funded ED salary throughout the duration of this study and provided feedbacks on the manuscript. ED and RC received joint funding to start the experiments in this study then ED received her own funding to complete the experimental work in this study. RC gave feedbacks on the manuscript before submission. ED has designed the experimental procedure, performed some experiments herself, supervised the first author undertaking most of the experiments, co-analyzed the results with the first author and wrote the manuscript. All authors contributed to the article and approved the submitted version.

## Supplementary material

Supplementary Figure 1 Androgen excess disrupt oestrous cyclicity. **(A,B)** Representative examples of oestrous cyclicity of a VEH **(A)** and PNA **(B)** mice after 21 days of vaginal smears. **(C)** Histogram of the frequency of each oestrous cycle phase in VEH and PNAF mice. Mean +/- SEM. ***p<0.001, multiple t-test. VEH = control female, PNAF = prenatally androgenized female mice modeling PCOS.

Supplementary Figure 2 Characterization of the progesterone receptor antibody Abcam 63605 in the adult female mediobasal hypothalamus. To our knowledge, while this antibody has been used in mouse uterine and ovaries tissues as well as on *in vitro* GnRH neurons from embryonic mouse explants, it has not yet been characterized in the mouse brain. The specificity of this PR antibody for PR has been demonstrated in uterine tissue lacking PR. The optimal concentration was determined by a dilution series and the sensitivity to detect PR in the mouse brain was demonstrated in brain tissue from mice with increasing circulating levels of oestradiol (OVX, diestrous, OVX+E2, n=2/group), as PR expression in the female brain is dependent upon oestradiol. We observed a slight increase in PR-immunoreactivity between the female mice in diestrous and the female OVX+E2 in hypothalamic brain regions where PR expression is expected (Supplemental Figure). A complete lack of PR-immunoreactivity was observed in OVX mice, consistent with a very low circulating levels of oestradiol. These data together support the specificity and sensitivity of this PR antibody to detect physiological differences in PR expression in the mouse brain. **(A)** Representative photomicrographs of the PR immunoreactivity in the mediobasal hypothalamus of an adult female mouse on diestrous. **(B)** Representative photomicrographs of the PR immunoreactivity in the mediobasal hypothalamus of an adult female mouse after at least 2-months of ovariectomy and estradiol replacement. (OVX + E). **(C)** Representative photomicrographs of the PR immunoreactivity in the mediobasal hypothalamus of an adult female mouse after 2-months of ovariectomy (OVX). Scale bar: Top panels 200 µm; Bottom panels 100 µm.
