# Supplementary figures and images for: Maternal androgen excess significantly impairs sexual behavior in male and female mouse offspring: Perspective for a biological origin of sexual dysfunction in PCOS
Source: Front Endocrinol (Lausanne). 2023 Feb 15;14:1116482. doi: 10.3389/fendo.2023.1116482 (PMC9975579; doi:10.3389/fendo.2023.1116482)

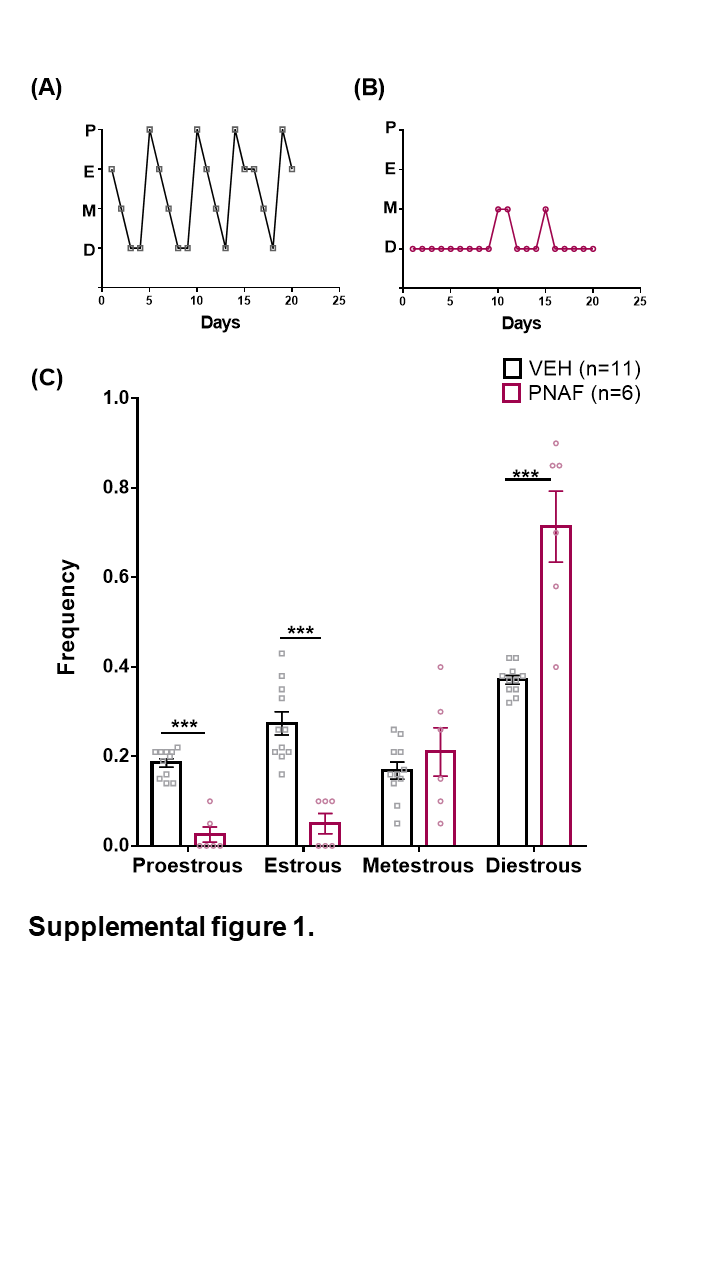

Supplement: Supplementary Figure 1 — Androgen excess disrupt oestrous cyclicity. (A,B) Representative examples of oestrous cyclicity of a VEH (A) and PNA (B) mice after 21 days of vaginal smears. (C) Histogram of the frequency of each oestrous cycle phase in VEH and PNAF mice. Mean +/- SEM. ***p<0.001, multiple t-test. VEH = control female, PNAF = prenatally androgenized female mice modeling PCOS. [file Image_1.tif]

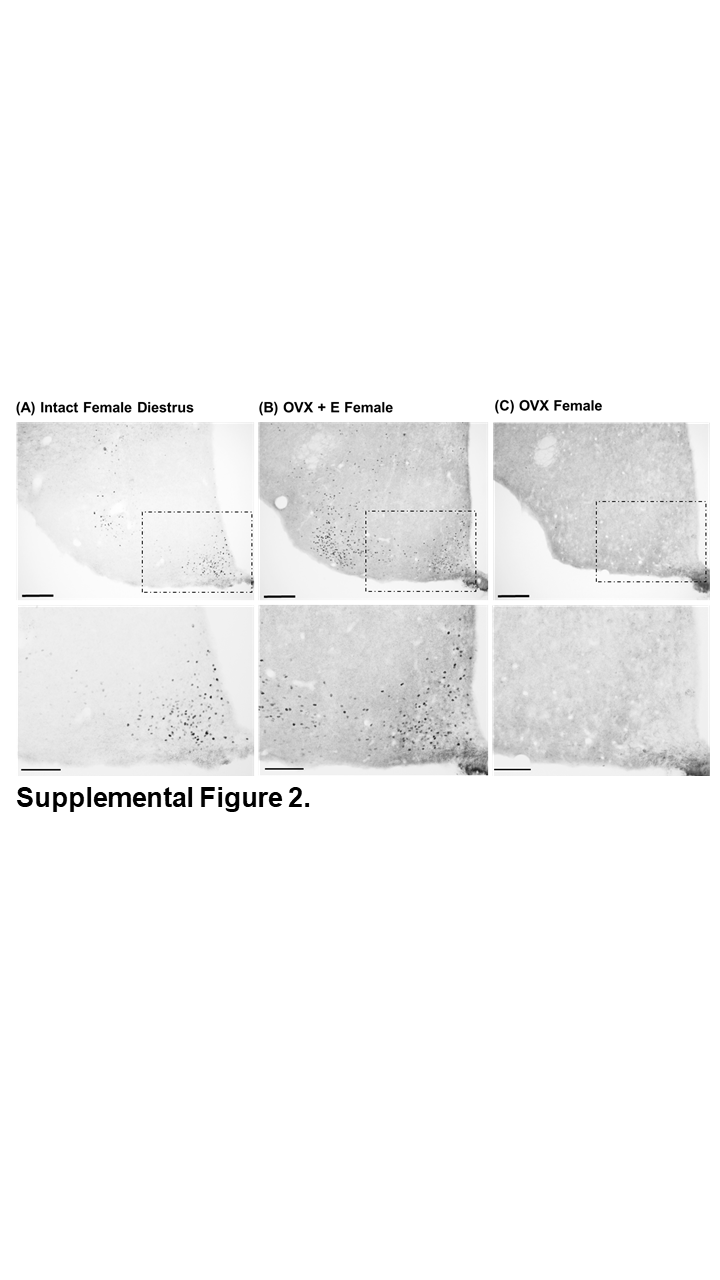

Supplement: Supplementary Figure 2 — Characterization of the progesterone receptor antibody Abcam 63605 in the adult female mediobasal hypothalamus. To our knowledge, while this antibody has been used in mouse uterine and ovaries tissues as well as on in vitro GnRH neurons from embryonic mouse explants, it has not yet been characterized in the mouse brain. The specificity of this PR antibody for PR has been demonstrated in uterine tissue lacking PR. The optimal concentration was determined by a dilution series and the sensitivity to detect PR in the mouse brain was demonstrated in brain tissue from mice with increasing circulating levels of oestradiol (OVX, diestrous, OVX+E2, n=2/group), as PR expression in the female brain is dependent upon oestradiol. We observed a slight increase in PR-immunoreactivity between the female mice in diestrous and the female OVX+E2 in hypothalamic brain regions where PR expression is expected (Supplemental Figure). A complete lack of PR-immunoreactivity was observed in OVX mice, consistent with a very low circulating levels of oestradiol. These data together support the specificity and sensitivity of this PR antibody to detect physiological differences in PR expression in the mouse brain. (A) Representative photomicrographs of the PR immunoreactivity in the mediobasal hypothalamus of an adult female mouse on diestrous. (B) Representative photomicrographs of the PR immunoreactivity in the mediobasal hypothalamus of an adult female mouse after at least 2-months of ovariectomy and estradiol replacement. (OVX + E). (C) Representative photomicrographs of the PR immunoreactivity in the mediobasal hypothalamus of an adult female mouse after 2-months of ovariectomy (OVX). Scale bar: Top panels 200 µm; Bottom panels 100 µm. [file Image_2.tif]
